# The effect of Ramadan fasting on cardiometabolic risk factors and anthropometrics parameters: A systematic review

**DOI:** 10.12669/pjms.315.7649

**Published:** 2015

**Authors:** Mohsen Mazidi, Peyman Rezaie, Owais Chaudhri, Ehsan Karimi, Mohsen Nematy

**Affiliations:** 1 Mohsen Mazidi, PhD. Key State Laboratory of Molecular Developmental Biology, Institute of Genetics and Developmental Biology, Chinese Academy of Sciences, Chaoyang, Beijing, China; 2 Peyman Rezaie, MSc. Biochemistry and Nutrition Research Center, School of Medicine, Mashhad University of Medical Science, Mashhad, Iran; 3 Owais Chaudhri, PhD. Diabetes and Endocrinology Service, Palmerston North Hospital, Palmerston North, New Zealand; 4 Ehsan Karimi, MSc. Biochemistry and Nutrition Research Center, School of Medicine, Mashhad University of Medical Science, Mashhad, Iran; 5 Mohsen Nematy, MD, PhD. Biochemistry and Nutrition Research Center, School of Medicine, Mashhad University of Medical Science, Mashhad, Iran

**Keywords:** Ramadan fasting, Cardiovascular risk, Body weight, High-density lipoprotein cholesterol, Body-mass index

## Abstract

Fasting during the month of Ramadan is a religious rituals of all healthy adult Muslims. However, there is no clear agreement on the effects of Ramadan fasting on cardiovascular disease. Comorbidities and factors such as age, gender, health status, daily duration of fasting, food intake before and after fasting may impact on a fasting individual’s cardiometabolic risk. This review was undertaken to assess the effects of Ramadan fasting on: the incidence of cardiovascular disease during the month of Ramadan; the clinical status of patients with stable cardiac disease; and any alterations in cardiometabolic risk profile.

**Methods:**

A systematic search was undertaken for studies that investigated the impact of Ramadan fasting on cardiovascular outcomes and risk factors. Electronic databases including MEDLINE, Scopus and Web of Knowledge were searched from 1982 up to 2014. The incidence of acute cardiac illness during Ramadan fasting was similar when compared to non-fasting days. Ramadan fasting is associated with elevations in high-density lipoprotein cholesterol (HDL-c), and reductions in low-density lipoprotein cholesterol (LDL-c) and total cholesterol (T-chol). However, the lipid profile of diabetic patients deteriorated significantly during Ramadan fasting. In addition, Ramadan fasting lowers body weight, body fat percentage and BMI (body mass index). However, the relationship between weight reduction and loss of body fat is not studied. The majority of patients with stable cardiac illness can opt for Ramadan fasting safely. However, the long term effects of Ramadan fasting on cardiovascular outcomes and risk factors remains uncertain, and the apparent discordant effects in individuals with and without diabetes mellitus merits further study.

## INTRODUCTION

There are approximately 1.6 billion Muslims worldwide. Ramadan, the ninth lunar month, is the holiest month in the Islamic calendar. During this month, millions of Muslims fast from dawn to sunset each day.^1^ Studies of fasting commonly distinguish between caloric restriction (CR), alternate-day fasting (ADF), and dietary restriction (DR). Ramadan fasting is considered similar to ADF because both incorporate alternating feast periods and fast periods.^2^

Areas of the world with large Muslim populations, such as the Middle East and South Asia, bear a heavy burden with respect to cardiometabolic disease and cardiovascular risk factors, such as diabetes mellitus.^3^,^4^ While doctrine exempts people with significant illness from the obligation of Ramadan fasting, many Muslim patients nevertheless have a strong desire to participate. This raises general concerns regarding safety. It also raises more specific questions regarding outcomes and necessary precautions if these patients complete fasting for the full month.

The literature on the effects of Ramadan fasting on cardiovascular outcomes and related factors are controversial. Fasting in Ramadan has been associated with both negative and positive effects in individuals with cardiovascular disease or cardiometabolic risk factors.^1^,^4^ In this review, we aimed to analyze the available literature on the effects of Ramadan fasting on cardiovascular outcomes in patients with a history of cardiovascular disease, as well as the effect of fasting on cardiometabolic risk factors such as circulating lipids.

## METHODS

A comprehensive search strategy was developed for original articles published in peer-reviewed journals, using terms related to the impact of Ramadan fasting on cardiovascular outcomes, risk factors and body composition. The following electronic databases were searched: MEDLINE, Scopus and Web of Knowledge from 1982 up to 2014.

Search was conducted using the search term: (“Ramadan” OR “Islamic fasting” OR “Muslim fast” OR “Islamic fast” OR “Islam fast”) AND (“Body weight” OR “weight reduction” OR “weight loss” OR “weight gain” OR “body mass index” OR “body composition” OR “BMI” OR “body water” OR “blood pressure” OR “waist circumference” OR “energy intake” OR “carbohydrate intake” OR “protein intake” OR “fat intake” OR “total cholesterol” OR “triglycerides” OR “high density lipoproteins” OR “low density lipoproteins” OR “very low density lipoproteins” OR “blood pressure” OR “pulse pressure” OR “cardiovascular diseases’’ OR “heart diseases” OR “stroke” OR “ heart failure” OR “ acute myocardial infarction” OR “cardiac care unit” OR “coronary heart diseases”).

Further articles were identified by examining the reference lists of identified articles and through the Science Citation Index. The search was limited to full papers published in the English language with no limit on the date of publication.

The identified studies were reviewed and articles were included in the analysis if they reported the effects of Ramadan fasting on cardiovascular outcomes, such as myocardial infarction, cardiac revascularization, heart failure or stroke, or on cardiometabolic risk factors such as body mass index (BMI), blood pressure (BP) or lipid profiles. Editorials and review articles were excluded, as were abstracts not associated with a full, original article. Studies concerned with the effects of fasting on groups not representative of a broad patient population (for example, athletes) were also excluded.

Following this initial process of filtering, the full text of the remaining papers was further reviewed. Case series, cohort studies and randomized studies were considered eligible for inclusion in the final analysis. Eligibility was determined by two reviewers (M.M. and M.S.), and differences of opinion settled following discussion.

## RESULTS

Just some studies were considered eligible for review. These studies may be grouped into those that considered cardiovascular outcomes, and those that considered risk factors alone.

### Cardiovascular Outcomes

No study was large enough in scope to consider the effects of Ramadan fasting on cardiovascular outcomes in disease naïve individuals, in people with established cardiovascular disease. However, a number of studies reported no effect of fasting during Ramadan on stroke, hospitalization for heart failure, acute myocardial infarction (AMI), the incidence of angina pain, coronary artery diseases and valvualr heart disease in patients with stable cardiovascular diseases.^5^-^8^

A retrospective study from Turkey appeared to show a deleterious effect of Ramadan fasting on the incidence of stroke in patients with diabetes mellitus.^9^ However, other studies^7^,^10^ have failed to show a difference.

Furthermore, although one study^11^ reported a decreased incidence of unstable angina and acute myocardial infarction during Ramadan fasting, this finding has not been replicated subsequently and collectively the results of this review showed no significant differences in the incidence of AMI or angina during Ramadan when compared to the rest of the year. For example, Chamsi-Pasha et al.^5^ carried out a prospective study on eighty-six outpatients with heart disease who were intending to fast. Forty-six patients (53%) had coronary artery disease, 23 patients (27%) had valvular heart disease, 13 patients (15%) had congestive heart failure and 4 patients (5%) were treated for arrhythmia. The results indicated that the effects of fasting during Ramadan on stable patients with cardiac disease were minimal.

**Table-I:** Cardiovascular disease and cardiometabolic risk factors.

Article				Subject’s characteristics			Outcomes								
	Year			Number & Sex				ACS				Stroke			
Author	Study	Publish	Location	M	F	Age (year)	HF	AMI	UA	VHD	Arrhythmia	Hypertensives	Diabetes	Hypertension	Diabetes
Temizhan A.	1991-1997	1999	Turkey	NM		NM	NM	↓		NM	NM	NM		NM	NM
Akhan J.	1991-1995	2000	Turkey	230000		>25	NM	NM		NM	NM	-		NM	NM
Comoglu	2003	2003	Turkey	126	137	63.9±11.2	NM	NM		NM	NM	↓	↑	NM	NM
Al Suwaidi J.	1991-2001	2004	Qatar	20 856		NM	NM	-	-	NM	NM	NM		NM	NM
Al Suwaidi J.	2004	2004	Qatar	5095	3351	NM	-	NM		NM	NM	NM		NM	NM
Ch-Pasha H.	1996	2004	KSA	54	32	56.3 (17-84)	-	-	-	-	NM	NM		NM	
Bener A.	1991-2003	2006	Qatar	335		56.99±13.9	-	NM		NM	-	-	-	-	

↑= a significant increasing; ↓= a significant decreasing; -= not significant change; NM= not mention in article. M= male; F= female; HF= heart failure; AMI=acute myocardial infarction; UA= unstable angina; VHD= valvular heart disease.

Similarly, in a larger (though retrospective) series, Al Suwaidi et al. considered hospitalizations for heart failure during Ramadan compared with the rest of the year. Data were collected on 8446 Qatari patients (5095 males and 3351 females) for a period of 10 years (January 1991 through December 2001). There appeared to be no significant difference in the number of hospitalization for heart failure while fasting in Ramadan when compared to the non-fasting months.^6^

### Cardiometabolic risk factors

### Blood pressure

Table-II summarizes the studies reporting the effects of Ramadan fasting on blood pressure. Three studies reported a significant reduction in systolic blood pressure during Ramadan compared to before Ramadan.^12^-^14^ with the exception of a further study^12^, which reported a significant increase in pulse pressure, the majority of the studies evaluated revealed that there was no difference in blood pressure during and before Ramadan.

**Table-II:** Effects of Ramadan fasting on blood pressure.

Article				Subject’s characteristics					Outcomes		
	Year			No. and Sex		Age	BW	BMI			
Author	Study	Publish	Location	M	F	(year)	(kg)	(kg/m)	SBP	DBP	PP
Habbal R.	1994-1997	1998	NM	27	72	56.7±9	NM	NM	-	-	NM
Perk G.	2001	2001	Israel	17		56.6±6.9	NM	NM	-	-	NM
Rahman M.	2004	2004	Bangladesh	20	0	NM	NM	NM	↓	NM	↑
Ural E.	2007	2008	Turkey	15	30	58±12	77.6±12.9	NM	-	-	NM
Shehab	2012	2012	UAE	70	32	38.7±10.5	82.9±14.6	28.1±4.4	↓	-	NM
Nematy M.	2012	2012	Iran	38	44	54±10	NM	NM	↓	-	NM

↑= a significant increasing; ↓= a significant decreasing; -= not significant change; NM= not mention in article.

M= male; F= female; BW= body weight; BMI= body mass index; SBP= systolic blood pressure;

DBP= diastolic blood pressure; PP= pulse pressure.

### Body weight and composition

Table-III summarizes the studies which reported the effects of Ramadan fasting on body weight and composition. In addition, Table-III shows a significant reduction in BMI and Waist circumference collectively.^12^-^18^

**Table-III:** Effects of Ramadan fasting on blood lipids and anthropometric parameters.

Article				Subject’s characteristics					Outcomes											
	Year			No. and Sex		Age	BW	BMI												
Author	Study	Publish	Location	M	F	(year)	(kg)	(kg/m^2^)	EI	CI	PI	FI	T-chol	TG	HDL	LDL	VLDL	BW	BMI	WC
Sulieman S.	1982	1982	UK and Sudan	20	4	30 (21-40)	69.5±2.3	NM	NM	NM	NM	NM	↑	-	NM	NM	NM	↓	NM	NM
Hallak M. H.	1988	1988	Syria	16	-	18-30	66.2±7.6	22.5±2.6	↑	NM	NM	NM	-	↓	↓	↑		↓		NM
Maislos M.	1992	1993	Israel	16	8	27 (18-45)	68±17	24.6±4.6	NM	NM	NM	NM	-	-	↑	-	-	-	-	NM
Adlouni A.	1997	1997	Moracco	32	-	NM	NM	NM	↑	NM	NM	NM	↓	↓	↑	↓	NM	↓	NM	NM
Maislos M.	1998	1998	Israel	32		NM	NM	NM	NM	NM	NM	NM	-	-	↑	-	-	NM	-	NM
Ziaee V.	2002	2002	Iran	41	39	20-35	62.4±11.6	21.2±4.5	NM	NM	NM	NM	-	-	↓	↑	-	↓	↓	NM
Afrasiabi A.	1997	2003	Iran	28	0	NM	NM	NM	↓	↓	↓	↓	↓	↓	-	↓	NM	NM	↓	NM
Afrasiabi A.	1998	2003	Iran	22	0	NM	NM	NM	↓	NM	NM	NM	-	↓	-	-	-	-	-	NM
Ch-Pasha H.	1996	2004	KSA	54	32	56 (17-84)	NM	NM	NM	NM	NM	NM	-	-	-	-	-	NM	NM	NM
Rahman M.	2004	2004	Bangladesh	20	0	NM	NM	NM	↑	NM	NM	↑	-	-	↑	-	-	↓	↓	NM
Unalacak M.	2007	2011	nm	10	0	NM	NM	NM	↓	NM	NM	NM	↓	↓	↑	↓	↓	↓	↓	NM
Barkia A.	2011	2011	Tunisia	19	6	42 (22-55)	NM	27.1±2.6	NM	NM	↑	↑	↑	-	↑	-	NM	NM	NM	NM
Sadiya A.	2011	2011	UAE	0	276	49±6	NM	34.63±3.29	-	NM	↓	↑	NM	NM	NM	NM	NM	↓	NM	↓
Shehab	2012	2012	UAE	70	32	38.7±10.5	82.9±14.6	28.1±4.4		NM	NM	NM	-	-	↑	↓	NM	↓	↓	↓
Nematy M.	2012	2012	Iran	38	44	54±10	NM	NM	-	-	-	-	↓	↓	↑	↓	↓	↓	↓	↓
Mirzaei B.	2012	2012	Iran	14	0	20.12±2.5	NM	70.61±18.4	NM	NM	NM	NM	↓	-	↑	↓	-	↓	NM	NM

↑= a significant increasing; ↓= a significant decreasing; -= not significant change; NM= not mention in article.M= male; F= female; BW= body weight; BMI= body mass index; EI= energy intake; CI= carbohydrate intake; PI= protein intake; FI= fat intake; T-chol= total cholesterol; TG= triglycerides; HDL= high density lipoproteins; LDL=low density lipoproteins; VLDL= very low density lipoproteins; WC= waist circumference.

### Lipid profile

In terms of metabolic profile, five studies reported a significant decrease in total circulating cholesterol (T-chol) during the month of Ramadan.^13^,^17^,^19^-^21^ This is in contrast with two studies which revealed a significant increase in T-chol during Ramadan.^22^,^23^

Six studies reported a significant reduction in triglycerides during this period.^13^,^15^,^17^,^19^,^20^,^24^ The effects of fasting on high density lipoprotein (HDL) levels was equivocal, with nine studies reporting a significant increase during Ramadan^12^-^14^,^17^,^19^,^21^,^22^,^25^,^26^ but two others reporting a decrease.^25^,^27^ A similar pattern was observed with low density lipoprotein (LDL). Six studies showed a significant decrease during Ramadan fasting.^13^-^15^,^17^,^19^,^21^ but a further two studies reported an increase in LDL.^24^,^16^ Most of the studies included in this review reported no effect on very low density lipoprotein (VLDL) levels^5^,^12^,^15^,^16^,^21^,^25^,^26^ although two did report a significant decrease in VLDL.^13^,^17^

## DISCUSSION

The high incidence of cardiovascular diseases in predominantly Muslim populations make an assessment of the effects of Ramadan on cardiovascular outcomes and parameters important. Broadly, the effects may be summarized as neutral or beneficial, although there are notable exceptions. For example, two studies in particular noted a deterioration in lipid profile during Ramadan fasting.^16^,^24^ Definitive answers are, however, difficult as long-term prospective data are lacking, and the literature comprises studies of generally small size. This latter limitation also confines outcome studies to populations in whom secondary prevention is considered, rather than primary prevention of cardiovascular disease.

Other limitations include the need to control for time of year. The timing of Ramadan moves throughout the year, in accordance with the phases of the moon. Therefore over a decade or so, one would anticipate fasting to have occurred throughout the year, and for seasonal influences on cardiovascular outcomes to be controlled. The short timeframe for many of the studies in the literature, however, introduces a complication and care must be taken in interpreting the results.

Many studies use individuals as their own controls, or are uncontrolled, introducing further limitations into the data. Comparison of fasting individuals with non-fasting individuals is problematic, as those who are exempted from fasting are generally exempted on the grounds of ill health. Randomization to fasting and non-fasting groups would be problematic, on the basis of interference with an individual’s strongly held beliefs.

Furthermore, the global reach of Islam as a religion makes wider application of results from one part of the world, where there are specific local cultural and eating habits, to another region, where the habits are different. In addition, many of the studies fail to account for common confounders, such as the effects of smoking, and physical activity.

Therefore, inconsistency in the results of different investigations on the influences of Ramadan fasting on cardiometabolic risk factors and anthropometrics parameters can be explained by the mentioned reasons and limitations regarding the studies related to Ramadan including diverse food habits and socioeconomic status of different sets of Muslims, differences in climate and fasting duration between counties, difference in the methods of studies, races and genders of the subjects as well as dissimilar life style in different countries, health status and medical history of each participant. Future research should therefore concentrate on the investigation of these factors.

Perhaps insights might be gained by considering parallel situations in other religions, or the comparison of CR (Calorie restriction) and DR (Dietary restriction) dietary regimens with ADF (Alternate-Day Fasting) regimens. Studies have revealed that calorie restriction with adequate nutrition may have beneficial effects on several metabolic and molecular factors that are modulating cardiovascular aging itself^27^ and also improve lipid profile, blood pressure and fasting blood sugar.^4^,^28^,^29^ Furthermore, it has been indicated that Short-term modified alternate-day fasting can be a novel dietary strategy for weight loss and cardioprotection in normal weigh and overweight adults.^30^, ^31^

Areas on which further study may be focused include the effects of Ramadan fasting on metabolic diseases including diabetes, the components of metabolic syndrome, kidney diseases and liver diseases like fatty liver disease.

## CONCLUSION

The literature does not support the association of Ramadan fasting with any change in incidence of cardiovascular illness, and the majority of cardiovascular patients can fast except those with diabetes mellitus according to the unfavorable changes in lipid profile and the specific dietary regimens of these patients. Generally, the effect of Ramadan fasting on cardiovascular risk factors appears to be neutral, but with some studies reporting benefit on lipid parameters, and others on BMI.
